# Necrotizing Fasciitis in a Patient With Signet-Ring Cell Gastric Adenocarcinoma

**DOI:** 10.7759/cureus.51198

**Published:** 2023-12-27

**Authors:** Lorenz Kristoffer Daga, Lovell Gatchalian

**Affiliations:** 1 Section of Gastroenterology, East Avenue Medical Center, Quezon City, PHL

**Keywords:** septic shock, complicated gastric cancer, necrotizing fasciitis, perforated gastric cancer, signet ring cell adenocarcinoma

## Abstract

Necrotizing fasciitis is an aggressive infection of the skin and soft tissues that requires prompt recognition and management. Immediate source control and adjunctive antibiotic therapy are the cornerstones of management. There are limited reported cases of necrotizing fasciitis related to gastrointestinal malignancy, including gastric cancer. This report describes the case of a 36-year-old male who developed left abdominal wall necrotizing fasciitis related to perforated gastric adenocarcinoma with signet-ring features. Signet-ring adenocarcinoma is associated with a more aggressive malignancy. The rapid progression of the infection leading to refractory shock and acute respiratory distress rendered the patient a poor candidate for surgical source control because of high surgical risk. Patient eventually expired. Immediate recognition of necrotizing fasciitis and perforated gastric cancer can prompt early surgical referral for definitive source control and gastric resection or repair.

## Introduction

Necrotizing fasciitis is a soft tissue infection characterized by friable fascia, gray exudates, and the absence of pus. The infection is usually polymicrobial and frequently develops among the elderly and those with comorbid illness and presents with soft-tissue edema, erythema, severe pain, tenderness, fever, and skin bullae or necrosis [1]. Severe sepsis can develop, especially in patients with immunosuppression, diabetes, malignancy, drug abuse, and chronic kidney disease [2]. At least 20% of cases are reported to be idiopathic [3]. The most common site of infection is limbs but infection involving the abdomen and perineum has been noted in 12.1% of cases. A 16.9 % overall mortality was noted in one study [4].

Laboratory tests including C-reactive protein, white blood cell count, hemoglobin, serum sodium, serum creatinine, and serum glucose can be used to assess the likelihood of patients having necrotizing fasciitis. Imaging such as computed tomography (CT) scan and magnetic resonance imaging (MRI) can help diagnose necrotizing fasciitis and identify other causes of infection. Prompt source control and adjunctive antimicrobial therapy are the mainstay in the treatment of necrotizing fasciitis [3].

Signet-ring cell gastric carcinoma is a histologic subtype of gastric adenocarcinoma with increasing incidence worldwide. It is significantly associated with a worse five-year survival rate compared to other types of gastric carcinoma (32% vs 45%) [5].

There are limited data describing necrotizing fasciitis in association with malignancy. This report aims to describe a case of necrotizing fasciitis associated with perforated gastric adenocarcinoma with signet-ring features.

## Case presentation

This is a case of a 36-year-old Filipino male, who presented with a six-month history of progressively enlarging painful epigastric mass associated with unintentional weight loss, intermittent post-prandial vomiting, and early satiety. There was no overt gastrointestinal bleeding noted. On physical examination, the epigastric mass was approximately 10 x 10 cm with poorly defined margins, non-movable, and nontender. There was no succussion splash or lymphadenopathy noted. 

**Figure 1:**
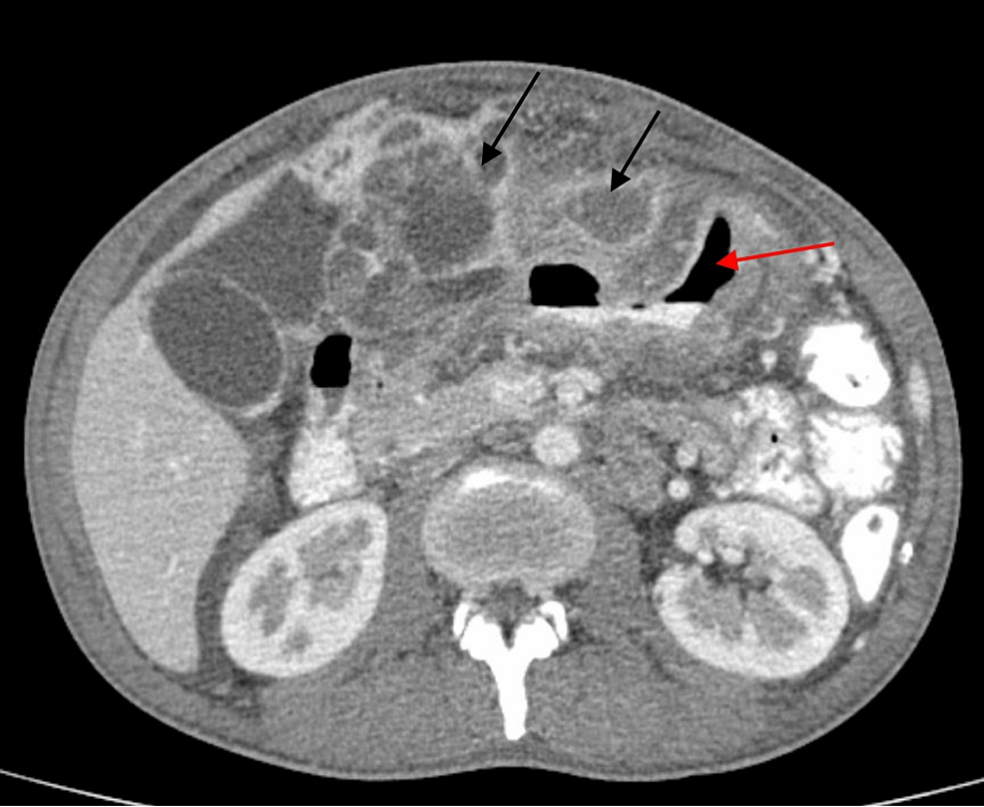
CT scan showing a gastric mass with areas of necrosis and diffuse irregular gastric wall thickening Black arrows show areas of necrosis within the gastric mass; Red arrow points to the gastric cavity.

A whole abdominal CT scan with intravenous, oral, and rectal contrast showed an 11.9 x 11.1 x 9.2 ill-defined necrotic gastric mass with enhancing peripheral nodular solid components and loculations with an invasion of the left liver lobe and compression of the pancreatic head and portal confluence. The said mass extended anteriorly to invade the left rectus sheath. The gastric wall had diffuse and irregular wall thickening. There were also enlarged retroperitoneal, perisplenic, peripancreatic, and perigastric lymph nodes and ascites (Figure 1).

**Figure 2:**
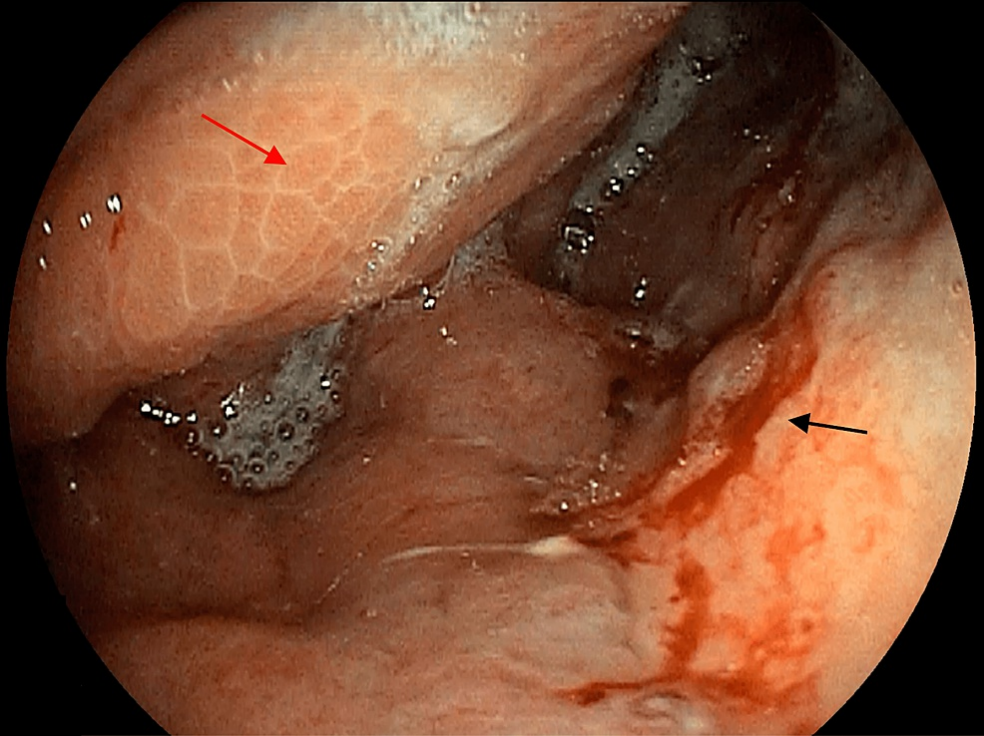
Poorly distensible stomach with diffuse friable mucosal thickening on endoscopy Black arrow shows friable gastric mucosa; Red arrow shows poorly distensible stomach.

Esophagogastroduodenoscopy revealed a poorly distensible stomach with circumferential friable thickened mucosa noted from the proximal stomach to the antrum (Figure 2). Biopsy samples were taken and histopathology showed adenocarcinoma with signet-ring features. 

**Figure 3:**
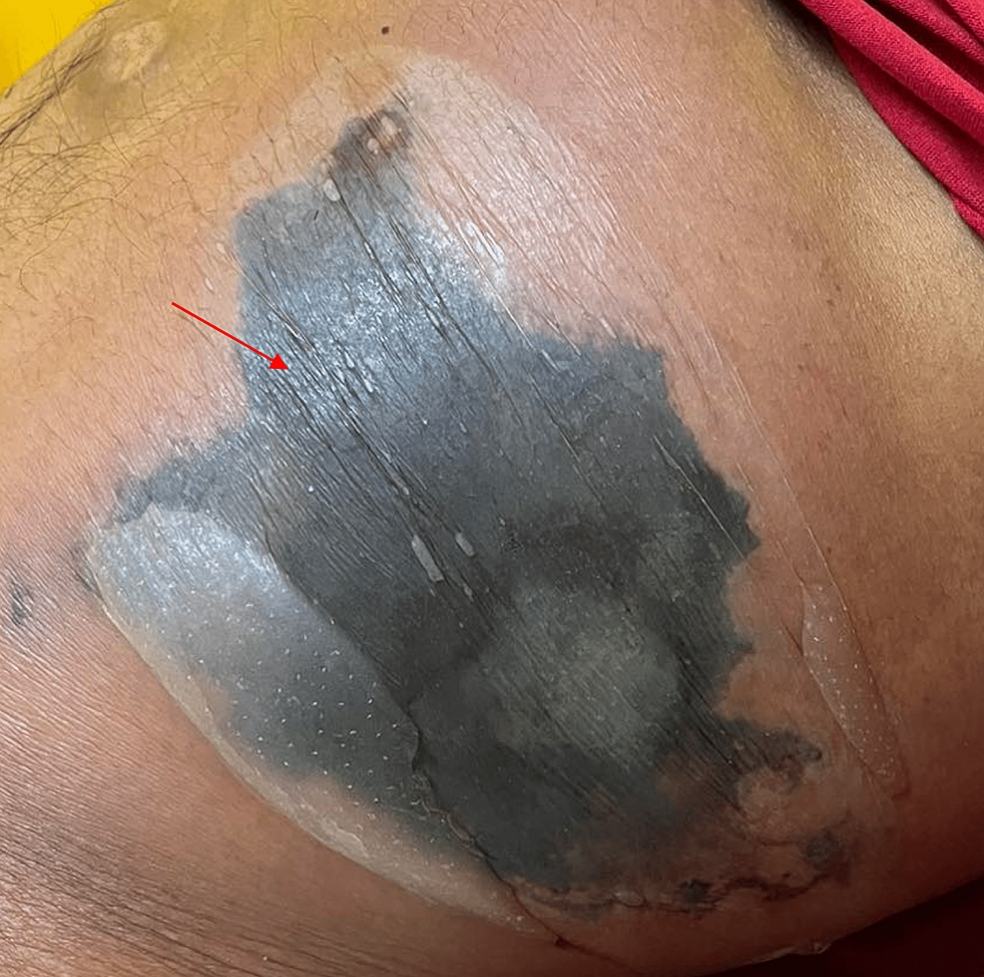
Bullae with surrounding erythema and necrosis in the left hemiabdomen Red arrow shows necrotic tissues.

**Figure 4:**
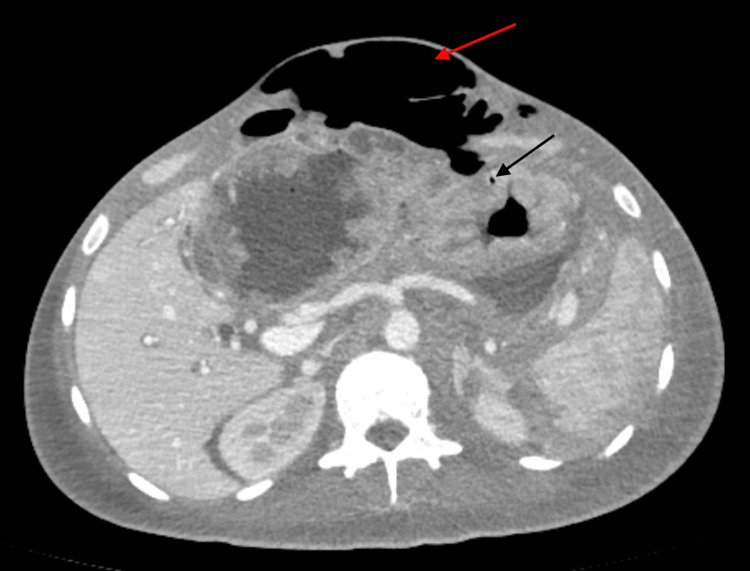
CT scan showing extensive soft tissue emphysema in the anterior abdominal wall Black arrow shows air within the gastric wall; Red arrow shows subcutaneous emphysema in the anterior abdominal wall.

The patient was advised chemotherapy with 5-fluorouracil, folinic acid, and oxaliplatin; however, bullae developed on the left hemiabdomen, which was associated with fever and abdominal distention. The bullae grew rapidly and eventually developed necrosis within two days (Figure 3). Repeat whole abdominal CT scan showed the development of pneumohydroperitoneum in the mid-abdomen and extensive soft tissue emphysema in the anterior abdominal wall extending to the pelvic region secondary to ruptured gastric mass (Figure 4). The assessment was necrotizing fasciitis secondary to perforated gastric mass. The patient was referred to infectious disease service and was started with ertapenem and clindamycin. Wound swab culture and blood culture showed no growth. Referral to general surgery was done for possible debridement and source control; however, four days after the onset of necrotizing fasciitis, the patient eventually developed acute respiratory distress syndrome and septic shock. Endotracheal intubation and mechanical ventilation were done and intravenous hydration and norepinephrine drip were started. The patient was unstable with high surgical risk and consent was not given for surgical management. Eight days after the onset of necrotizing fasciitis, patient developed refractory shock despite norepinephrine, dobutamine, and dopamine infusions, and eventually expired.

## Discussion

The incidence of gastric signet-ring cell carcinoma (SRCC) is increasing in Asia, the United States of America, and Europe and is found in 8-30% of gastric cancers. It is diagnosed at a younger age compared to non-SRCC and is found more frequently in the middle of the stomach and at a more advanced stage. Peritoneum is the most frequent site of metastasis. This histologic type is also associated with more aggressive cancer with a lower R0 resection rate. [5] The signet-ring features in our case could have contributed to the locally aggressive nature of the gastric adenocarcinoma of our patient, which lead to invasion of the left liver lobe and abdominal wall. 

Necrotizing fasciitis is a life-threatening soft tissue infection that requires prompt source control. There was a reported case of perineal necrotizing fasciitis, which was more often described in relation to lower gastrointestinal lesions, that developed in a gastric cancer patient treated with ramucirumab [6]. Necrotizing fasciitis, in that case, was concluded to be related to ramucirumab and not as a direct complication of gastric cancer compared to our case, which was related to perforated gastric cancer. 

There is a paucity of literature describing the occurrence of necrotizing fasciitis secondary to perforated gastric cancer. Most of the reported cases regarding the development of necrotizing fasciitis related to gastrointestinal malignancy are related to perforated colon cancer [7-8]. 

Perforated gastric cancers are most commonly located in the distal stomach and are usually presented as stage III or stage IV disease. These cases most commonly present as peritonitis and not as necrotizing fasciitis. These occur in less than 5% of all gastric cancers with an overall mortality of 2-46%. Surgical resection is the goal of treatment for perforated gastric cancers [9]. Compared to our case, the bulk of the tumor was located in the middle of the stomach and the perforation of the tumor presented as necrotizing fasciitis. Because of rapid clinical deterioration and hemodynamic instability, the patient was assessed to have a high risk to develop surgical complications; hence, surgical management, including resection, was not performed. 

Principles of treatment for necrotizing fasciitis include source control, antimicrobial therapy, and monitoring. Early and complete debridement is essential as antimicrobial therapy alone is associated with almost 100% mortality. Only the complete removal of infected tissue can lead to control of infection and recovery. Adjunctive antimicrobial therapy should be given until all infected tissues are removed and with noted improvement in the patient’s clinical condition [3]. Surgical management was essential in our case as the patient had two indications for prompt surgery: perforated gastric cancer and necrotizing fasciitis. A high index of suspicion, immediate recognition of these clinical entities, and prompt surgical referral were important in this case, especially when there was still a window for surgical management when the patient was still more clinically stable and had a lower risk for surgical complications.

## Conclusions

This case presented a patient with rapidly progressing necrotizing fasciitis associated with perforated gastric adenocarcinoma with histologic signet-ring features. Although broad spectrum antibiotics were started, the lack of source control could have led to rapid progression to infectious complications such as refractory septic shock and ARDS and the patient’s eventual demise. Early recognition of necrotizing fasciitis and perforated gastric cancer can prompt early surgical referral for source control and gastric resection or repair.
